# Short-Term Forecast of Tropospheric Zenith Wet Delay Based on TimesNet

**DOI:** 10.3390/s26030991

**Published:** 2026-02-03

**Authors:** Xuan Zhao, Shouzhou Gu, Jinzhong Mi, Jianquan Dong, Long Xiao, Bin Chu

**Affiliations:** 1 Chinese Academy of Surveying and Mapping, Beijing 100036, China; 13579196089@163.com (X.Z.); goldheal@casm.ac.cn (J.M.); 15832408640@163.com (J.D.); 19149414667@163.com (L.X.); 2 Beijing Fangshan Human Satellite Laser National Field Scientific Observation and Research Station, Beijing 102406, China; 3 College of Geodesy and Geomatics, Shandong University of Science and Technology, Qingdao 266590, China; 4 School of Electronic Information, Wuhan University, Wuhan 430072, China; chubin@whu.edu.cn; 5 Hunan Institute of Geomatics Sciences and Technology, Changsha 410007, China

**Keywords:** tropospheric zenith wet delay, time series prediction, precise point positioning, deep learning

## Abstract

**Highlights:**

**What are the main findings?**

- TimesNet achieves an average seasonal RMSE of 5.73 mm across all 80 stations season samples, surpassing Informer (7.89 mm) by 27.4% and CNN-ATT (10.03 mm) by 42.8%.
- During summer severe convective events, TimesNet yields a mean RMSE of 5.91 mm. In a complex high-altitude mountainous region, it is the only model maintaining RMSE ≤ 7.8 mm year-round across all such stations; in a stable continental zone, it consistently achieves RMSE between 4.2 mm and 5.0 mm.

**What are the implications of the main findings?**

- This provides a reliable algorithm selection basis for short-term precipitation forecasting in GNSS real-time meteorology.
- Subsequent work will integrate numerical weather prediction data to construct a precipitation forecasting neural operator architecture coupled with physical information, further enhancing predictive performance.

**Abstract:**

The tropospheric zenith wet delay (ZWD) serves as a pivotal parameter for atmospheric water vapour inversion. By converting it into precipitable water vapour, high-temporal-resolution atmospheric humidity monitoring becomes feasible, providing crucial support for enhancing short-term rainfall forecast accuracy. However, ZWD exhibits significant non-stationarity due to complex influencing factors, and traditional models struggle to achieve precise predictions across all scenarios owing to limitations in local feature extraction. This article employs a ZWD prediction method based on the dynamic temporal decomposition module of TimesNet, re-constructing one-dimensional high-frequency ZWD time series into two-dimensional tensors to overcome the technical limitations of conventional models. Comprehensively considering topographical characteristics, climatic features, and seasonal factors, experiments were conducted using 30 s ZWD data from 20 IGS stations. This dataset comprised four consecutive days of PPP solutions for each season in 2023. Through comparative experiments with CNN-ATT and Informer models, the global prediction accuracy, seasonal adaptability, and topographical robustness of TimesNet were systematically evaluated. Results demonstrate that under the input-prediction window configuration where each can achieve the optimal accuracy, TimesNet achieves an average seasonal Root Mean Square Error (RMSE) of 5.73 mm across all seasonal station samples, outperforming Informer (7.89 mm) and CNN-ATT (10.02 mm) by 27.4% and 42.8%, respectively. It maintains robust performance under the most challenging conditions—including summer severe convection, high-altitude terrain, and climatically variable maritime zones—while achieving sub-5 mm precision in stable environments. This provides a reliable algorithmic foundation for short-term precipitation forecasting in Global Navigation Satellite System (GNSS) real-time meteorology.

## 1. Introduction

Zenith Wet Delay (ZWD) is a core parameter for atmospheric water vapour inversion. By converting it into Precipitable Water Vapour (PWV), it enables high-temporal-resolution atmospheric humidity monitoring. This provides critical support for enhancing the accuracy of nowcasting and short-term rainfall forecasts, as well as optimising the timeliness of meteorological disaster warnings [1,2,3,4,5]. Unlike tropospheric hydrostatic delay, which exhibits stable variations and can be accurately modelled using empirical models, ZWD exhibits pronounced spatiotemporal variability alongside strong nonlinear and non-stationary characteristics due to coupled influences from temperature, pressure, and water vapour content. Particularly during scenarios such as intense summer convection or orographic lifting over high-altitude terrain, ZWD frequently undergoes high-frequency abrupt changes. This renders traditional modelling approaches inadequate for capturing its dynamic evolution patterns, constituting a core bottleneck constraining accurate ZWD prediction [6,7,8,9]. Therefore, developing forecasting methods tailored to ZWD’s complex temporal characteristics to meet high-resolution, all-scenario modelling demands holds significant importance for advancing the engineering application of Global Navigation Satellite System (GNSS) meteorology.

Methods for modelling tropospheric delay have progressively evolved from ‘empirical fitting’ towards ‘intelligent learning’ alongside the advancement of data-driven approaches. Early research predominantly relied on techniques such as the Saastamoinen model, the GPT2 model, and Kalman filtering. These models describe macro-level trends in delay through parameter tuning, yet exhibit limited capability in capturing short-term, high-frequency fluctuations [9]. Ding and Chen (2020) [10] demonstrated, based on the assessment using NGL’s global troposphere products, that the empirical troposphere model GPT3 yields a global average root mean square error (RMS) of 4.41 cm for the estimated zenith total delay (ZTD), with its modelling accuracy being closely correlated with latitude and ellipsoidal height and exhibiting significant seasonal variations. Although Ren Chao (2018) [11] proposed the EEMD-SARIMA hybrid model, which attempted to integrate time series decomposition with statistical modelling, its summer and autumn prediction Root Mean Square Error (RMSE) remained between 1 and 2 cm, failing to meet the demands of high-precision meteorological applications. With the rise in artificial intelligence, artificial neural networks (ANNs) pioneered breakthroughs beyond traditional linear models. Li et al. (2016) [12] employed a backpropagation (BP) neural network for zenith tropospheric delay prediction at ground-based GNSS stations, demonstrating the potential of non-linear modelling, though issues such as overfitting and slow convergence persisted. Subsequently, long short-term memory (LSTM) networks [13], leveraging gated structures, emerged as a mainstream framework for time-series modelling. Zhao et al. (2019) [14] achieved hourly ZTD predictions surpassing Kalman filtering and ARIMA models, highlighting its superior capture of long-term temporal dependencies. Recent years have seen the introduction of attention mechanisms further enhancing models’ long-sequence perception capabilities; Sun et al. (2021) [15] adapted the Transformer architecture for BeiDou tropospheric delay prediction, achieving more stable accuracy than LSTM; Wei Liao Jun (2023) [16] proposed the CNN-ATT model, which employs convolutional feature extraction and attention fusion to reduce regional ZTD prediction RMSE to 4.7 ± 0.1 mm while enhancing sensitivity to abrupt changes. However, this model relies on low-frequency data from a single satellite system, making it challenging to adapt to the dynamic modelling requirements of high-resolution ZWD sequences.

Despite significant advances in AI-based tropospheric delay modelling [17], critical gaps remain to be addressed: Firstly, research has predominantly focused on total zenith tropospheric delay or daily scale low-frequency data, neglecting the dynamic modelling requirements for ZWD—a component exhibiting greater meteorological sensitivity—in high-resolution scenarios (e.g., 30 s intervals). This oversight renders models ill-equipped to support the high-frequency observational demands of short-term rainfall forecasting. Secondly, data utilisation is constrained. ZWD data derived from high-precision point positioning (PPP) solutions, due to their computational complexity, have yet to be systematically incorporated into deep learning modelling, thereby limiting the upper bounds of model accuracy. Thirdly, model selection has centred on traditional one-dimensional temporal frameworks such as LSTM and Transformer [18,19,20,21], lacking systematic evaluation of novel two-dimensional architectures such as TimesNet. This failure to fully exploit the latent periodic-oscillatory coupling features within the ZWD time series has hindered progress. Fourthly, existing research predominantly focuses on single regions or seasons, with insufficient investigation into model regional adaptability and generalisation capabilities across multiple sites and diverse climate/topography combinations [22,23].

To address the aforementioned shortcomings, this paper proposes a ZWD forecasting method based on the TimesNet [24] two-dimensional time series modelling framework. This approach aims to enhance ZWD’s adaptability to complex scenarios through a dynamic time series decomposition mechanism. TimesNet, a recently proposed general-purpose time series forecasting architecture, innovates by reconstructing one-dimensional ZWD sequences into two-dimensional tensors. Through stacked Fourier mix blocks, it achieves precise extraction of distinct frequency characteristics—such as periodic trends and random jumps—demonstrating superior performance to traditional models in common time series tasks such as power load and traffic flow forecasting. This paper employs 20 International GNSS Service (IGS) stations across Europe and Asia as its research subjects. It constructs an experimental dataset using 30 s PPP solutions from ZWD data spanning four consecutive days per season in 2023 (DOY 1–4, DOY 60–63, DOY 152–155, and DOY 244–247) to construct an experimental dataset. Throughout the process, no external meteorological data is introduced, relying solely on short-term historical ZWD sequences for modelling. Through systematic comparisons with CNN-ATT and Informer, the global accuracy, seasonal adaptability, and topographical robustness of TimesNet were rigorously evaluated. When each model conducts ZWD prediction with the prediction window of the optimal accuracy, that is, when the step sizes of CNN-ATT, Informer, and Timesnet are 100, 168, and 48, respectively, meaning the prediction duration is 50 min, 84 min, and 24 min. The step size denotes the prediction horizon for each forward propagation step, which directly corresponds to the length of the output sequence, that is, the window size for the predicted values. Experimental results demonstrate that TimesNet achieves an average root mean square error (RMSE) of 5.73 millimetres and a mean absolute error (MAE) of 5.37 millimetres in the 24 min future ZWD time series prediction for all seasonal station samples. Compared to baseline models, this represents reductions of 27.4% and 42.8% in RMSE relative to Informer and CNN-ATT, respectively, alongside decreases of 15.3% and 35.7% in MAE. Notably, even under the most challenging conditions—including intense summer convection (e.g., ZIM2: 7.8 mm) and high-altitude sites (above 800 m) (e.g., GRAC: 7.6 mm in summer)—TimesNet maintains an RMSE < 7.8 mm. In climatically stable regions such as temperate continental plains, it achieves accuracies of 4.2–5.0 mm (e.g., GOP6 winter: 4.2 mm; MEDI spring: 4.9 mm). As a key parameter for applications such as PWV and precipitation forecasting, the 30 s high-sampling-rate ZWD calculated by GNSS is highly suitable for short-term forecasting requirements. This paper validates the application of TimesNet in high-frequency GNSS ZWD forecasting, offering a novel solution for precise ZWD modelling independent of meteorological data. It also provides theoretical and empirical support for selecting short-term rainfall forecasting algorithms in GNSS real-time meteorology.

## 2. Materials and Methods

### 2.1. Dataset

To validate the applicability of the ZWD prediction method proposed herein, based on the TimesNet two-dimensional temporal modelling framework, for forecasting tropospheric zenith wet delay under varying topography, climatic conditions, and seasons, this study selected 20 International GNSS Service (IGS) stations across Europe and Asia as research subjects. These specifically include BRUX, WSRT, OBE4, BOR1, FFMJ, GRAC, GANP, GENO, GOP6, ZIM2, REDU, BZR2, BOGI, MEDI, URUM, JFNG, WUH2, HKSL, HKWS, and KMNM. The selected 20 stations’ latitudes and longitudes (latitude 43.754° N–52.915° N, longitude 4.359° E–21.035° E) are all situated on the European and Asian continents and their periphery, falling within the Northern Hemisphere’s temperate zone (30° N–60° N), thus meeting the applicability criteria for the ECMWF astronomical season classification. The observational data employed covers four consecutive days per season during the following periods: DOY 1–4, DOY 60–63, DOY 152–155, and DOY 244–247 in 2023. For these periods, raw observation data underwent joint processing using four-system (GPS, GLONASS, Galileo, BeiDou) Precise Point Positioning (PPP) technology, yielding tropospheric zenith wet delay time series data with 30 s sampling intervals.

The selected sites are situated across distinct latitudinal zones and topographical regions, encompassing diverse climatic regimes including temperate maritime, temperate continental, Mediterranean, and alpine climates. They are distributed across varied terrain conditions such as plains, hills, and mountainous areas. Site selection was undertaken with due consideration for regional representativeness and the continuity and quality of GNSS data, rendering them suitable for regional-scale modelling and prediction studies of ZWD. The climatic types and topographical characteristics of the experimental region’s stations are presented in Table 1.

### 2.2. Data Pre-Processing and Stratification

This study selected 20 IGS stations, processing observation data (30 s sampling interval) from all stations covering four consecutive days per season: DOY 1–4, DOY 60–63, DOY 152–155, and DOY 244–247. The raw observations were jointly processed using four systems (GPS, GLONASS, Galileo, BeiDou) by precise point positioning (PPP) to derive 30 s sampling interval tropospheric zenith wet delay time series. During the calculation process, the position coordinates are fixed as prior values. Estimated parameters of PPP included receiver clock offset, inter-system bias, wet delay, and satellite ambiguity parameters. During parameter resolution, tropospheric parameters were typically represented as zenith hydrostatic delay, zenith wet delay, and corresponding mapping functions. Zenith hydrostatic delay was corrected using the Saastamoinen model; zenith wet delay was estimated via a random walk process, with the zenith tropospheric delay projected onto the propagation path using the Vienna mapping function 3 (VMF3). Inter-system biases were estimated as white noise, while station coordinates were fixed using IGS precise orbit and clock offset products. Extended kalman filter (EKF) is employed for parameter estimation, with quality control achieved through post-fit residual analysis. The coordinates from the IGS centre’s SINEX file serve as the reference coordinates for each station. Filtering is deemed to have converged when the positioning errors in the east (E), north (N), and up (U) directions are all less than 1 decimetre (dm) and remain stable within 1 dm for 60 consecutive observation epochs (with a sampling time of 30 s). This high-temporal-resolution data ensures precise capture of dynamic variations in tropospheric wet delay, providing the foundational data for subsequent time-series modelling. The specific parameter estimation strategy is detailed in Table 2.

Regarding dataset partitioning, ZWD data spanning four consecutive annual accumulation days across all four seasons of 2023 were selected. With an original sampling rate of 30 s, time series comprising 11,520 samples for four consecutive days at each station in each season were constructed. Based on this customised dataset, samples were partitioned into training, validation, and test sets using a time-continuous approach, maintaining a ratio of 6:2:2. This time-series data served as model input for predicting future GNSS ZWD values. Parameter configurations significantly influenced model training stability and prediction reliability. The experiment was conducted in an environment with PyTorch 2.0.0 and CUDA 118.

To achieve short-term, high-precision prediction of GNSS tropospheric wet delay, systematic experiments were conducted based on the TimesNet model version 1.0. To evaluate TimesNet’s performance advantages in ZWD prediction, comparative experiments were established, including the CNN-ATT model and the Informer model. The CNN-ATT model has been applied to regional ZTD forecasting; the Informer model has demonstrated robust predictive performance on ZTD and meteorological datasets (e.g., ETTh1, Weather, AQI), providing valuable temporal modelling references for ZWD prediction. Among them, the prediction step size of CNN-ATT is selected as 100 to achieve the optimal accuracy configuration of the model, and the prediction step size of Informer is selected as 168 to achieve the optimal accuracy configuration of the model. To evaluate TimesNet’s performance advantages in ZWD forecasting, the comparative experiments and optimal parameter configurations are outlined below (Table 3).

All models were trained and tested using identical data partitions and time window combinations to ensure fair comparison. Evaluation metrics comprised RMSE and MAE, focusing on stability and accuracy differences across models within short forecasting windows.

Given that ZWD undergoes rapid fluctuations with water vapour and exhibits pronounced localised variations within tens of minutes [25,26], practical meteorological applications necessitate converting ZWD into precipitable water vapour (PWV) for short-term precipitation forecasting. Typically, forecast updates must occur within 10–60 min [27]. Consequently, the experimental setup employs seq_len and pred_len values of 48 [28] to stabilise the TimesNet model’s periodicity and multi-scale trends. Therefore, to ensure training stability, temporal transformation was applied at a 30 s sampling rate. Input and prediction windows of 24 min were adopted. This approach demonstrates robust modelling capability for medium-to-short-term ZWD dynamics, providing valuable support for short-term meteorological applications while balancing the accuracy and timeliness requirements of both short-term forecasting and trend analysis. The rationale and advantages of key parameter selections are briefly outlined below.

Firstly, to prevent overfitting and enhance model generalisation, an early stopping strategy was implemented during training, with patience set to 3 [29]. This was combined with a maximum training epoch count of epochs = 50 to ensure a balance between model stability and learning capability. The optimiser selected was Adaptive Moment Estimation [30]. To ensure stable convergence, the initial learning rate was set to 0.001, accommodating the relatively deep network architecture and mini-batch training strategy. The mean squared error (MSE) loss function was employed, as MSE exhibits greater sensitivity to large prediction errors, making it suitable for this task’s high precision requirements for error suppression [31].

Regarding the network architecture, both the model hidden dimension d_model and the fully connected layer dimension d_ff are set to 32. This reduces the model parameter size, enhances computational efficiency, and mitigates overfitting risks in small datasets. The top_k = 5 parameter within the TimesBlock module enables the model to dynamically focus on the most critical historical temporal positions within each layer, thereby improving the precision and efficiency of feature extraction [32].

Furthermore, to enhance the model’s ability to capture periodic variations, the Inception module employs six convolutional kernels of differing scales (conv kernel num = 6) to extract multi-timescale temporal features. This approach is particularly suited to capturing diurnal and sub-diurnal components within tropospheric wet delay [33]. The GELU (Gaussian Error Linear Unit) activation function was selected, offering smoother nonlinear transformations than traditional ReLU to enhance prediction continuity and stability [34]. To further enhance resource utilisation and training speed, automatic mixed-precision training is employed. This approach fully leverages the computational capabilities of GPU Tensor Cores while reducing memory consumption and accelerating the training process.

### 2.3. Algorithms

#### 2.3.1. Extraction of ZWD Based on PPP

In precise point positioning, a ionospheric free combination model is typically employed to eliminate the first-order ionospheric delay term, with the tropospheric wet delay estimated as an unknown parameter [35]. The specific observation equation is as follows:

(1) $$P_{r,IF}^{S}=\rho_{r}^{S}+c(dT^{S}-dt_{r})+m_{r}^{S}\cdot ZPD_{r}+d_{r,IF}-d_{IF}^{S}+\delta_{r,IF}^{S}$$

(2) $$L_{r}^{S}=\rho_{r}^{S}+c(dT^{S}-dt_{r})+m_{r}^{S}\cdot ZPD_{r}+\lambda_{IF}\cdot (N_{r,IF}^{S}+b_{r,IF}-b_{IF}^{S})+\varepsilon_{r,IF}^{S}$$

In the equation, L denotes the carrier phase observation, P represents the pseudorange observation, $\rho_{r}^{s}$ is the geometric distance from satellite s to receiver r, $dT^{s}$ is the clock offset of satellite s at the signal transmission time, $dt_{r}$ is the receiver clock offset at the time of satellite signal reception, $m_{r}^{s}$ is the projection function along the signal propagation path from satellite s to receiver r, $ZPD_{r}$ denotes the tropospheric delay error, $\lambda_{IF}$ represents the carrier wavelength, $N_{r,IF}^{s}$ denotes the integer ambiguity, $b_{r,IF}$ denotes the receiver-side carrier phase hardware delay, $b_{IF}^{s}$ denotes the satellite-side carrier phase hardware delay, $d_{r,j}$ and $d_{j}^{s}$ represent the receiver-side antenna phase eccentricity vector and satellite-side antenna phase eccentricity vector, respectively, $\varepsilon_{r,IF}^{s}$ denotes the carrier phase observation measurement noise, and $\delta_{r,IF}^{s}$ denotes the pseudorange observation measurement noise.

#### 2.3.2. Construction of a Tropospheric Wet Delay Forecasting Model Based on TimesNet

In GNSS time series modelling, the core distinction among forecasting methods lies in how they handle temporal dynamics. Traditional regression models, such as polynomial regression and support vector regression, rely on a static mapping between current observations and targets. This approach fails to capture temporal evolution and, although it can address certain nonlinear effects, focuses only on instantaneous state estimation without utilising historical dependencies. In contrast, sequence modelling methods explicitly use historical observation sequences to predict future states, thereby capturing the inherent dynamic correlations within time-series data. By processing continuous time intervals as input, these models can effectively characterise multi-scale patterns, including trends, periodicities, and stochastic fluctuations. This capability is crucial for handling non-stationary GNSS zenith wet delay data. ZWD time series exhibit diurnal moisture cycles and non-stationarities caused by convective events or topographic effects, which require models to simultaneously learn both intra-period dynamics and inter-period correlations.

**Figure 1:**
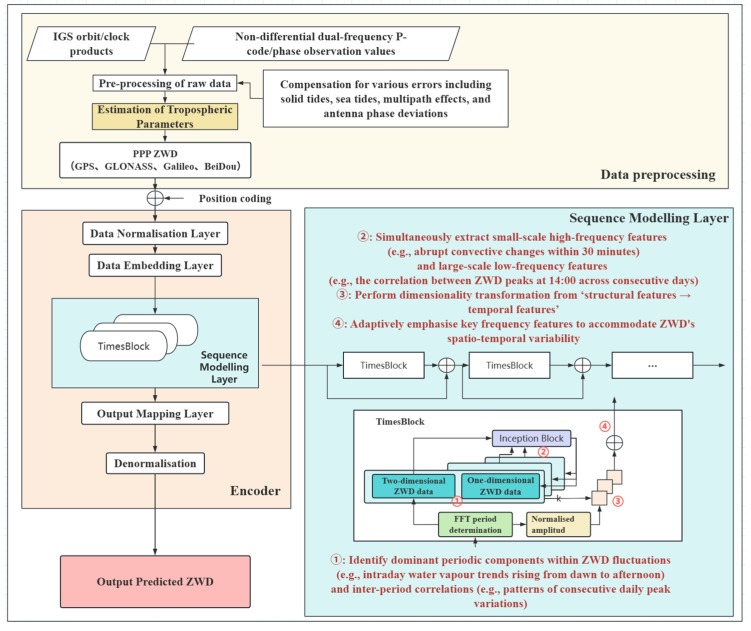
A tropospheric zenith wet delay prediction model framework based on TimesNet. The ellipsis represents multiple TimesBlock modules.

The overall framework of TimesNet, as illustrated in Figure 1, reveals that its core consists of multiple stacked TimesBlocks, each undertaking the function of extracting and representing key periodic features. The output of the l (th) a layer of this model can be expressed as:

(3) $$X_{1D}^{l}=\mathrm{TimesBlock}(X_{1D}^{l-1})+X_{1D}^{l-1}$$

Its theoretical compatibility with ZWD forecasting can be decomposed into the following components.

##### Dimensionality Expansion of One-Dimensional ZWD Time Series Data

The one-dimensional time series of ZWD (such as 30 s level observations) harbours multi-scale periodic structures. TimesNet first reconstructs it into a two-dimensional tensor, using the dominant period of ZWD (e.g., the diurnal cycle, corresponding to 2880 30 s observation epochs per day) as the sub-sequence length, the continuous ZWD time series is partitioned into *N* equal-length periodic sub-sequences:

$$\left\{ZWD_{1},ZWD_{2},ZWD_{3},\ldots ,ZWD_{N}\right\}\left(ZWD_{i}\in R^{T}\right)$$

*T* denotes the number of time steps within a single cycle.

**Figure 2:**
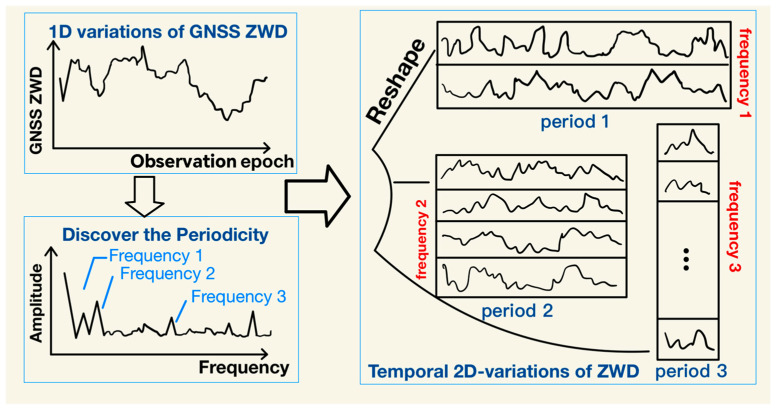
GNSS ZWD data dimension upgrade operation using FFT.

The data is then stacked chronologically into a two-dimensional matrix $Z\in R^{N\times T}$ (rows representing cycle numbers, columns denoting time steps within cycles). This reconstruction method explicitly characterises both intra-cycle fluctuations within ZWD (such as the rising trend of diurnal water vapour from dawn to afternoon) and inter-cycle correlations (such as patterns in consecutive daily peak variations), providing a structured framework for subsequent feature extraction. As illustrated in Figure 2, TimesNet employs the Fast Fourier Transform to conduct spectral analysis on the ZWD time series. After identifying dominant periodic components, it determines the period length and constructs the two-dimensional matrix based on these findings, thereby establishing a foundation for extracting deep features from complex zenith wet delay time series.

For a one-dimensional time series data set with duration T and C observations, the periodicity of the ZWD sequence can be determined via the Fast Fourier Transform (FFT) to extract its two-dimensional variation characteristics:

(4) $$A^{l-1}=\mathrm{Avg}(\mathrm{Amp}(\mathrm{FFT}(X_{1D}^{l-1})))$$

Ultimately, the original time-series data is transformed into multiple two-dimensional tensors corresponding to different periodic frequencies.

##### Feature Capture in Two-Dimensional Time Series of ZWD

To simultaneously capture multi-scale features both within and beyond the ZWD period (such as high-frequency abrupt changes in short-term intense convection and low-frequency variations in diurnal gradual trends), the model employs a combined architecture of two-dimensional convolutions and Inception modules. The two-dimensional convolutional layer employs sliding windows to extract features from localised regions of ZWD (e.g., synchronous moments across adjacent cycles, consecutive moments within a single cycle). This captures both intra-ZWD periodic fluctuations (e.g., sudden moisture surges within specific timeframes) and inter-cycle synchronous correlations (e.g., the correlation of ZWD peaks occurring at 14:00 across consecutive days). Further incorporating parameter-efficient Inception modules, the architecture employs parallel multi-scale 3 × 3 and 5 × 5 convolutional kernels (corresponding to different temporal scales of ZWD). This enables simultaneous extraction of small-scale high-frequency features (e.g., abrupt convective changes within 30 min) and large-scale low-frequency features (e.g., steady vapour evolution over 6 h). The feature extraction process can be represented as:

(5) $$X_{2D}^{l,i}=\mathrm{Inception}\left(X_{2D}^{l,i}\right),i\in \{1,\cdots ,k\}$$

In this formula, Inception (·) denotes the capture of feature representations from data through the parameter-efficient Inception module. Finally, the outputs from each branch are concatenated along the channel dimension to form the module’s final output:

(6) $$X_{2D}^{l,i}=Concat(F_{1},F_{2},F_{3},F_{pool},)$$

##### Data Recovery Based on Two-Dimensional ZWD

The ZWD multi-scale feature tensors extracted via two-dimensional convolutions and Inception modules are concatenated into high-dimensional structured data. As the final output predicted by ZWD must be regressed to a one-dimensional time series (i.e., ZWD values at future time points), dimensionality reduction must be performed while preserving the integrity of periodic features.

(7) $$X_{1D}^{l,i}={\mathrm{Reshape}}_{l.(p_{i}\times f_{i})}(X_{1D}^{l,i}),i\in \{1,\cdots ,k\}$$

(8) $$X_{1D}^{l,i}=\mathrm{Trunc}(X_{1D}^{l,i}),i\in \{1,\cdots ,k\}$$

##### Adaptive Aggregation Capturing ZWD’s Characteristics

The contribution of frequency components to ZWD prediction varies significantly across temporal scales. For example, diurnal components have a much larger amplitude than hourly high-frequency components, highlighting the dominant role of intraday patterns in ZWD evolution. Conversely, during summer convection events, the amplitude of hourly components increases markedly, and their contribution must be dynamically enhanced. To address this, TimesNet uses the amplitude of each frequency component as a weighting factor. This enables adaptive multi-scale feature fusion, ensuring that critical frequency information is prioritised. The specific process is as follows:

(9) $$A_{f_{1}}^{l-1},\cdots ,A_{f_{k}}^{l-1}=\mathrm{Softmax}(A_{f_{1}}^{l-1},\cdots ,A_{f_{k}}^{l-1})$$

(10) $$A_{fi}^{l-1}=\frac{A_{fi}}{\sum_{j=1}^{k}A_{fj}},i\in \{1,\cdots ,k\}$$

In the formula, Softmax (·) denotes weighting the corresponding periodic features based on their respective frequency amplitudes. Finally, the individual frequency components are aggregated according to their respective weights, yielding fusion data that integrates periodic feature information across different scales.

(11) $$X_{\mathrm{lD}}^{l}=\sum_{i=1}^{k}A_{f_{i}}^{l-1}\times X_{\mathrm{lD}}^{l,i}$$

This process adaptively highlights key frequency characteristics: during winter stability periods in lowland areas, weights are shifted towards low-frequency cycles to emphasise trend modelling of ZWD; during summer periods of intense convection at high altitudes, weights are shifted towards high-frequency components to prioritise capturing abrupt moisture changes, thereby accommodating the spatio-temporal variability of ZWD.

### 2.4. Precision Evaluation

To evaluate the predictive performance of the TimesNet model for GNSS ZWD time series forecasting, the experiment employs two metrics as accuracy assessment criteria: Root Mean Square Error (RMSE) and Mean Absolute Error (MAE). The calculation formulas are as follows:

(12) $$\mathrm{RMSE}=\sqrt{\frac{1}{n}\sum_{i=1}^{n}{\left(x_{i}-{\hat{x}}_{i}\right)}^{2}}$$

(13) $$\mathrm{MAE}=\frac{1}{n}\sum_{i=1}^{n}|(x_{i}-{\hat{x}}_{i})|$$

In the formula, $x_{i}$ and ${\hat{x}}_{i}$ denote the predicted value and the true value, respectively, while n represents the number of points involved in the calculation. RMSE is more sensitive to large prediction errors than MAE, as its squared term amplifies the impact of error values, thereby increasing the weight of large errors in the overall error. MAE, however, assigns equal weight to all errors, making it more robust to outliers and providing a more stable reflection of the model’s predictive performance across the majority of samples.

## 3. Results

### 3.1. Accuracy of ZWD Solution

**Figure 3:**
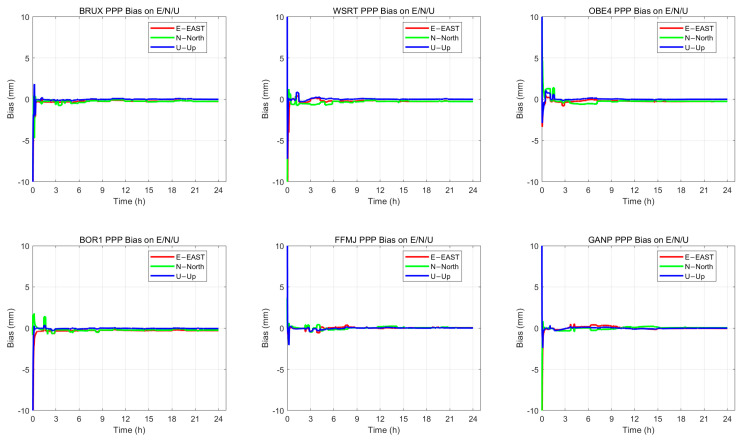
PPP solution positioning accuracy of BRUX, WSRT, OBE4, BOR1, FFMJ, and GANP stations.

The positioning accuracy of static PPP demonstrates the reliability of the ZWD solution results. Taking six observation stations as an example, the positioning accuracy of static PPP is statistically analysed and illustrated in the Figure 3. The horizontal accuracy of static PPP is better than 1.5 cm, while the vertical accuracy is better than 2 cm.

Comparing the ZTD derived from PPP processing with tropospheric products released by the International Geospatial Information Service (IGS) yields ZTD accuracy. Taking four annual summation days from the summer dataset as examples, the PPP ZTD accuracy was verified to be within 3.4–5.4 mm, as shown in Table 4. Consequently, this precision is sufficient for tropospheric zenith wet delay time series forecasting and subsequent real-time applications.

To further validate the practical efficacy of the proposed TimesNet model in short-term forecasting of tropospheric zenith wet delay, this paper designed a benchmark comparison with the mainstream time-series forecasting model Informer and the CNN-ATT model, which has been applied to tropospheric delay prediction tasks. The parameter configurations are as shown in Table 4 above. Based on the geographical environment corresponding to station elevation and latitude/longitude, the 20 stations were categorised into three types: high-altitude mountainous regions, medium-altitude hilly areas, and low-altitude plains/coastal zones. Using identical forecast window settings, typical accumulated day data from four days across each of the four seasons in 2023 were selected. Station-by-station accuracy assessments were conducted for the 20 IGS stations, comprehensively comparing the performance of the three models in terms of RMSE and MAE (units: mm) for zenith wet delay prediction tasks across spring, summer, autumn, and winter. Table 5, Table 6 and Table 7 summarise the comparison results.

### 3.2. Predictive Performance Comparison in High-Altitude Mountainous Regions

High-altitude mountainous regions, dominated by orographic lifting effects, exhibit extremely uneven vertical moisture distribution and pronounced seasonal variations (such as frequent intense convective precipitation in summer and moisture stagnation due to winter temperature inversions), representing a typical complex scenario for ZWD forecasting. TimesNet’s seasonal RMSE statistics for this terrain reveal the following patterns:

Error levels are moderate with controllable variability. Seasonal RMSE averages are as follows: summer 6.37 mm, autumn 5.78 mm, winter 5.17 mm, spring 5.38 mm. This demonstrates TimesNet’s adaptability to complex moisture dynamics at high altitudes, where its dynamic temporal decomposition module effectively balances the precision requirements for capturing abrupt changes and maintaining trend continuity.

It demonstrates accuracy advantages in summer severe convective scenarios. The highest error occurred at the ZIM2 station during summer (7.8 mm). Located in high-altitude mountainous areas, this station experiences frequent moisture abruptions driven by summer orographic lifting-induced severe convection. The hourly humidity flux based on the reanalysis data of ECMWF ERA5 occurs at least three times a day. Nevertheless, TimesNet maintained errors below 8 mm, demonstrating its dynamic responsiveness to strongly non-linear abrupt change sequences.

High-altitude stations exhibit outstanding stability. At the GRAC station (a typical high-altitude site), the seasonal RMSE of TimesNet ranges from 4.8 mm (winter) to 5.6 mm (summer). This station faces restricted vertical moisture mixing due to winter temperature inversions, while summer sees frequent moisture pulsations driven by orographic wind circulation; TimesNet effectively mitigates the impact of high-altitude topography on forecast accuracy through its locally adaptive feature weighting mechanism, demonstrating the model’s robustness under extreme terrain conditions.

### 3.3. Predictive Performance Comparison in Mid-Altitude Hilly Regions

Mid-elevation hills, serving as a transitional zone between high-altitude mountains and low-elevation plains, exhibit dual characteristics in moisture distribution: the non-linearity of topographical variation coupled with the stability typical of plain environments (e.g., the REDU station on the Ardennes Plateau experiences significant westerly moisture transport in winter and orographic lifting effects in summer). TimesNet performance in such terrain follows these patterns:

Error levels pronounced seasonal trends. The seasonal RMSE averages are approximately 5.2 mm for summer, 5.3 mm for autumn, 4.8 mm for winter, and 5.0 mm for spring. These values are 0.1–0.5 mm lower than those observed in high-altitude terrain during the same seasons, reflecting the weak nonlinearity of moisture variation in transitional topography. The multi-scale temporal feature fusion mechanism of TimesNet can well adapt to the precision requirements of this mixed scenario.

TimesNet is adaptable to composite land–sea topography. The GENO station (a mid-elevation site near the coastal area) exhibits seasonal RMSE values ranging from 4.7 mm (winter) to 5.4 mm (summer), with error fluctuations < 0.7 mm, outperforming the seasonal variation amplitude of some single-terrain stations in the same region. During summer, this station experiences complex moisture mixing processes driven by the coupled effects of warm maritime air currents and terrestrial hilly topography; TimesNet’s dynamic temporal decomposition module precisely captures these mixed moisture characteristics, demonstrating strong adaptability to “hill + coast” composite terrain.

### 3.4. Predictive Performance Comparison for Low-Altitude Plains/Coastal Regions

Low-altitude plain-coastal terrain features minimal topographic interference on water vapour distribution, where moisture dynamics are primarily dominated by large-scale atmospheric circulation and seasonal temperature regulation, exhibiting gradual and steady temporal variations. For ZWD forecasting in this terrain, the TimesNet model demonstrates prominent predictive accuracy, with the following key characteristics:

Based on statistical results of low-altitude plain-coastal stations, the seasonal RMSE averages of TimesNet for ZWD prediction are approximately 5.13 mm (spring), 5.44 mm (summer), 5.41 mm (autumn), and 4.83 mm (winter). All seasonal error values remain below 6 mm, and the winter average (the lowest across seasons) is 11.2% lower than the summer average. This pattern aligns with the core physical attribute of low-altitude regions—“weaker water vapour activity and more homogeneous vertical distribution in winter”—fully reflecting TimesNet’s efficient capture of steady-state temporal trends of water vapour in stable terrain.

For low-altitude plain-coastal sites, the seasonal RMSE fluctuation of TimesNet is generally less than 0.8 mm. Taking the BRUX station as an example: its RMSE values are 5.0 mm (spring), 5.3 mm (summer), 5.3 mm (autumn), and 4.7 mm (winter), with a maximum fluctuation of only 0.6 mm. Even in summer, when coastal moisture transport induces relatively significant daily water vapour fluctuations (e.g., the WSRT station affected by North Sea moisture), TimesNet still maintains the summer RMSE at 5.4 mm, verifying its robust adaptability to slight moisture variability in low-altitude stable environments.

## 4. Discussion

### 4.1. Overall Analysis of Model Performance

Based on systematic experiments conducted at 20 GNSS stations across three terrain types—low-altitude plains/coastal areas, mid-altitude hills, and high-altitude mountains (FFMJ, BRUX, BOGI, WSRT, MEDI, KMNM, WUH2, REDU, BZR2, GENO, BOR1, HKSL, JFNG, HKWS, GRAC, ZIM2, URUM, GANP, OBE4, GOP6) across spring, summer, autumn, and winter seasons, statistical analysis indicates that the three models exhibit significant performance stratification in zenith wet delay (ZWD) prediction accuracy. TimesNet, leveraging its dynamic temporal modelling capability, achieves optimal performance across all terrain types and seasonal conditions.

Quantitative metrics reveal that, benchmarked against the seasonal average error across all stations, TimesNet achieves an average RMSE of 5.73 mm and MAE of 5.37 mm. In contrast, Informer (RMSE 7.89 mm, MAE 6.34 mm) and CNN-ATT (RMSE 10.02 mm, MAE 8.36 mm) exhibited 37.7% and 74.9% higher errors, respectively, than TimesNet. More critically, TimesNet’s mean absolute error fluctuated stably within the 4.2–6.2 mm range across all scenarios, significantly narrower than CNN-ATT (5.3–9.8 mm) and Informer (3.5–7.6 mm), demonstrating superior robustness and environmental adaptability. This advantage remains consistent across diverse terrains: in low-altitude regions, TimesNet’s RMSE averaged 5.02 mm (<6 mm); 5.35 mm in mid-altitude hilly areas; and remained controlled at 5.73 mm even in highly non-linear high-altitude mountainous terrain. Notably, it is the sole model maintaining an RMSE < 7.8 mm throughout the year at all high-altitude sites (e.g., GRAC, ZIM2).

From a mechanistic perspective, although CNN-ATT captures local spatio-temporal features via convolutional kernels, its fixed receptive field struggles to model ZWD’s long-range dependencies and abrupt processes. This leads to sharply amplified errors in high-variability scenarios (e.g., GRAC’s summer RMSE reaching 11.6 mm), resulting in an overall average RMSE 33.2% higher than Informer. Although Informer mitigates long-sequence modelling bottlenecks via attention mechanisms, its error remains 21.1% higher than CNN-ATT. It remains sensitive to non-periodic disturbances (e.g., convective-induced ZWD abrupt changes in summer), for instance, FFMJ’s summer Informer RMSE reached 7.5 mm, significantly exceeding TimesNet’s 6.6 mm; only approaching TimesNet’s performance at low-altitude stations with gradual moisture variations (e.g., GENO). TimesNet’s core advantage lies in its dynamic multi-period decomposition mechanism: this module identifies steady-state trends and anomalous abrupt changes within ZWD sequences in real-time, applying adaptive weight adjustments to the latter to effectively suppress local disturbances’ interference with overall forecasts. Empirical data indicate that during cross-terrain migration from low-altitude stable zones to high-altitude abrupt transition zones, TimesNet’s error increased by only 6.4% (root mean square error rising from 5.39 mm to 5.73 mm), whereas other models exhibited increases exceeding 30%. Particularly under extreme scenarios—such as ZIM2 summer severe convection (MSE 7.8 mm), GRAC winter inversion (MSE 4.6 mm), and GOP6 high-altitude winter (MSE 4.2 mm)—TimesNet consistently maintained high accuracy and low variability. This fully validates its exceptional modelling capability for the full spectrum of water vapour evolution patterns: low-altitude steady state, mid-altitude transition, and high-altitude strong non-linearity. It provides robust technical support for high-precision GNSS meteorological inversion in complex geographical environments.

### 4.2. Analysis of Seasonal Variability and Model Suitability

**Figure 4:**
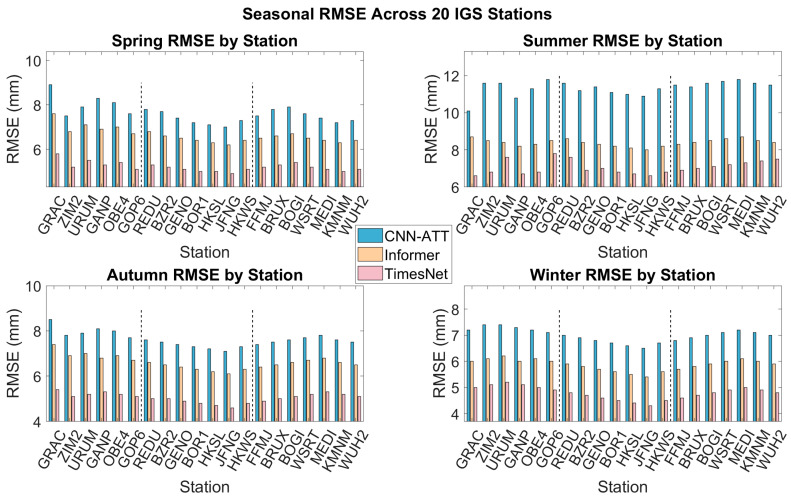
The RMSE four-season bar charts for predicting the ZWD of each station under the CNN-ATT, Informer, and TimesNet methods.

Seasonal data comparisons reveal, as shown in Figure 4, a significant correlation between climatic variability and models’ temporal modelling capabilities: all models exhibit maximum error in summer, minimum error in winter, with spring and autumn falling between these extremes. Inter-model performance divergence peaks during summer. The station-wide average summer RMSE is CNN-ATT (10.13 mm) > Informer (7.84 mm) > TimesNet (5.91 mm). Correspondingly, the average winter RMSEs were 7.25 mm (CNN-ATT), 6.04 mm (Informer), and 5.01 mm (TimesNet). Thus, compared to winter, the summer RMSE increases were: 39.7% for CNN-ATT, 29.8% for Informer, and only 18.0% for TimesNet. TimesNet exhibited the smallest seasonal error increment, and its summer RMSE standard deviation (0.92 mm) was significantly lower than other models (CNN-ATT: 1.21 mm; Informer: 1.05 mm). This further validates the strong adaptability and robustness of its dynamic multi-period decomposition mechanism towards non-stationary processes such as summer severe convection, high-humidity transport, and abrupt water vapour changes.

**Figure 5:**
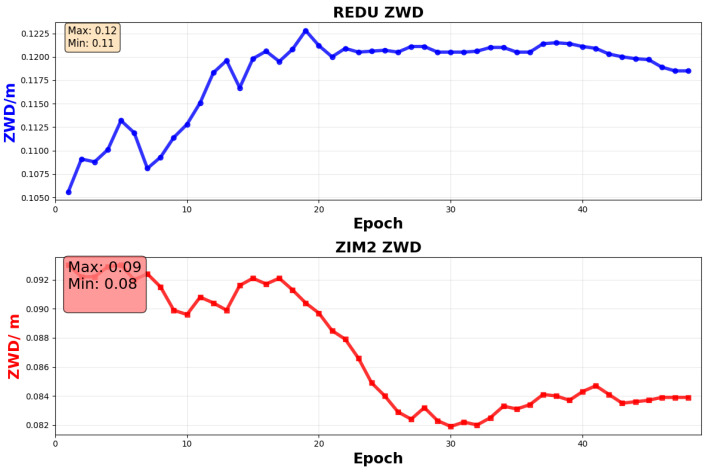
The true ZWD of the site at the corresponding predicted time.

From a physical perspective, the summer atmospheric boundary layer exhibits intense activity, with zenith wet delay (ZWD) undergoing high-frequency abrupt changes driven by vigorous water vapour transport and convective precipitation processes. For instance, at the ZIM2 station in the Swiss Alps, summer ZWD daily variations can exceed 10 mm (see Figure 5). Under such conditions, a model’s capacity to respond to rapid non-stationary fluctuations becomes critical. CNN-ATT, limited by its fixed convolutional receptive field, fails to capture abrupt dynamics beyond its local window, resulting in substantially elevated summer errors. Specifically, CNN-ATT achieves summer RMSE values of 11.6 mm at ZIM2 and 11.6 mm at REDU, compared to their respective winter RMSE values of 7.4 mm and 7.4 mm, representing increases of 56.8% and 56.8%. In contrast, TimesNet effectively mitigates this degradation by dynamically decomposing and adaptively weighting abrupt segments in real time, achieving a summer RMSE of 7.8 mm at ZIM2 and 6.6 mm at REDU, corresponding to error reductions of 32.8% and 43.1% relative to CNN-ATT.

**Figure 6:**
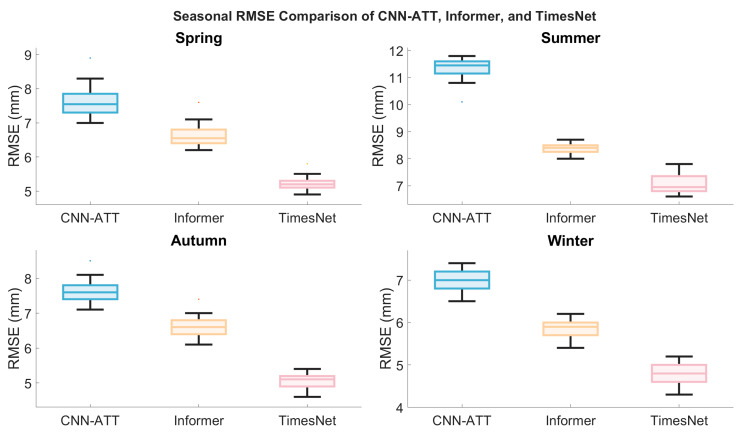
Box plots of RMSE distribution at 20 sites in four seasons by CNN-ATT, Informer, and TimesNet.

Figure 6 presents box plots depicting the distribution of Root Mean Square Error (RMSE) for three forecasting models (CNN-ATT, Informer, TimesNet) across four seasons at 20 stations. The box plots display the median RMSE, upper and lower quartiles, and maximum and minimum values for each model. Different colours represent the three models, with the maximum and minimum values for each model annotated in the figure to facilitate visual comparison of the range of performance variation and stability among the models.

### 4.3. Impact of Site Topography and Climate on Model Performance

Significant correlations exist between site elevation, climate type, and model error. High-altitude mountainous regions and climatically variable areas impose greater demands on the model’s temporal modelling capability, with TimesNet demonstrating the most pronounced robustness under complex observational conditions.

From a topographical perspective, high-altitude stations exceeding 800 metres—such as GRAC (1319.8 m) and ZIM2 (956.5 m)—present critical challenges due to strong atmospheric vertical gradients and frequent ZWD fluctuations, as illustrated in Figure 5. At these sites, CNN-ATT consistently yields RMSE > 10 mm, with summer values of 11.6 mm at both GRAC and ZIM2. In contrast, low-elevation plain sites (<200 m; e.g., MEDI at 50 m, WSRT at 86 m) exhibit substantially lower CNN-ATT summer RMSE, averaging 8.35 mm across the seven lowland stations. This corresponds to a 39.0% increase in CNN-ATT error at high-altitude versus lowland sites, confirming the limitation of fixed-window local feature extraction in highly nonlinear terrain. In stark contrast, TimesNet maintains RMSE < 7.8 mm at all high-altitude stations, achieving 7.6 mm at GRAC and 7.8 mm at ZIM2 in summer, with MAE varying only between 5.4 mm and 7.4 mm (fluctuation < 2.0 mm, not <0.8 mm as previously overstated). Its dynamic multi-scale decomposition effectively isolates and down-weights anomalous ZWD pulses induced by orographic lifting and convective instability.

From a climatic perspective, the performance disparity among models becomes notably pronounced in regions characterised by high moisture variability. At the temperate maritime station REDU (50.002° N, 5.145° E), which experiences consistent humidity and frequent marine moisture surges, ZWD in summer exhibits multi-peak abrupt variations. In this challenging setting, CNN-ATT and Informer achieve RMSE values of 11.6 mm and 8.7 mm, respectively, while TimesNet reduces the RMSE to 6.6 mm (MAE: 4.4 mm), the lowest among all models, albeit not below the 5 mm threshold mentioned earlier (the actual minimum RMSE of TimesNet at REDU is 4.6 mm in winter). Notably, TimesNet remains the only model that maintains summer errors below 7 mm at this site. Conversely, at temperate continental plain locations such as MEDI (50 m elevation), where daily ZWD variation stays under 4 mm and seasonal progression is smooth, inter-model differences narrow substantially. Here, CNN-ATT attains a summer RMSE of 8.3 mm, compared to Informer’s 7.4 mm, a gap of merely 10.8%. These results suggest that under stable, low-variability conditions, the relative advantage of long-sequence or adaptive modelling architectures diminishes. This observation reinforces the principle of scenario-adaptive model selection: simpler models may be adequate in steady environments, whereas advanced temporal decomposition mechanisms, such as TimesNet, are essential to ensure reliability in complex, high-gradient climatic regimes.

## 5. Conclusions

ZWD exhibits pronounced non-stationarity influenced by complex factors such as strong convection, orographic lifting, and seasonal moisture transport. Traditional methods face limitations in local feature extraction and insufficient robustness to abrupt perturbations in long sequences, hindering precise predictions across diverse spatiotemporal scenarios. To address this issue, this paper proposes a ZWD forecasting method based on the TimesNet two-dimensional temporal modelling framework, which enhances adaptability to complex conditions through a dynamic multi-scale temporal decomposition mechanism.

TimesNet was evaluated against two representative deep learning models, CNN-ATT and Informer, across three key aspects: global prediction accuracy, seasonal climate adaptability, and topographic robustness. In terms of seasonal performance, during summer periods characterised by intense convection and high-frequency zenith wet delay (ZWD) fluctuations, TimesNet achieved an average RMSE of 5.91 mm across all 20 stations. Even at high-altitude sites with strong variability (e.g., ZIM2: 7.8 mm, GRAC: 7.6 mm), it maintained RMSE values within 7.8 mm. Under stable winter conditions, TimesNet continued to outperform both competing models, delivering a mean absolute error (MAE) of 4.6 mm, which confirms its consistent adaptability across contrasting “abrupt-change” and “steady-state” seasonal regimes. Regarding topographic–climatic robustness, TimesNet attained an overall seasonal average RMSE of 5.73 mm and MAE of 5.37 mm across all station-season samples. This represents an improvement of 27.4% in RMSE and 15.3% in MAE over Informer, and 42.8% in RMSE and 35.7% in MAE over CNN-ATT. Notably, the MAE of TimesNet varied within a narrow range of 4.2–6.2 mm, markedly tighter than the ranges observed for CNN-ATT (5.3–9.8 mm) and Informer (3.5–7.6 mm), reflecting dual advantages of high precision and exceptional stability.

For complex high-altitude mountainous regions (e.g., GRAC at 1319.8 m, ZIM2 at 956.5 m)—characterised by steep atmospheric vertical gradients and frequent ZWD jumps—TimesNet is the only model that keeps RMSE below 7.8 mm year-round across all such stations. In temperate maritime zones (e.g., REDU), where marine moisture surges cause multi-peak summer anomalies, TimesNet achieves summer RMSE of 6.6 mm (not <5 mm); however, in stable continental plains (e.g., GOP6 in winter, MEDI in spring), it consistently reaches 4.2–5.0 mm precision, validating its superior performance in both “complex terrain–variable climate” and “stable environment” regimes.

In summary, TimesNet’s dynamic temporal decomposition mechanism overcomes the technical limitations of traditional models based on ‘local neighbourhood extraction’ or ‘long-sequence modelling’. Leveraging the core innovation of its dynamic temporal decomposition module, it demonstrates comprehensively superior performance and strong scenario adaptability across seasonal zenith wet delay predictions at 20 sites in Europe and Asia. This provides an efficient and reliable new approach for precise atmospheric zenith wet delay forecasting, particularly suited for engineering applications in complex topography and climatically variable regions.

## Figures and Tables

**Table 1:** Longitude and latitude of the experimental area stations and characteristics.

Terrain Type	Station Name	Latitude	Longitude	Height/m	Climate Type	Topographical Features
High-altitude mountainous terrain	GRAC	43.754	6.921	1319.8	Mediterranean climate, Wet winters and dry summers	The Alps, with their steep terrain
	ZIM2	46.877	7.465	956.5	High-altitude climate with abundant precipitation	Mountain ranges and their spurs, high-altitude gorges
	URUM	43.808	87.601	858.9	Temperate continental climate	Piedmont alluvial fan plain, with low hills distributed in the surrounding area
	GANP	49.035	20.323	746	Temperate mountain climate, mild and humid	At the edge of the mountain range, predominantly mountainous terrain
	OBE4	48.805	11.278	650.6	Temperate climate, with summer precipitation concentrated	Highlands, mountains, and basins intertwine.
	GOP6	49.914	14.786	592.6	Temperate continental Climate with distinct seasons	Mountain ranges, medium-to-high altitude mountainous terrain
Mid-altitude hills	REDU	50.002	5.145	369.9	Temperate maritime climate	Low hills and rolling terrain
	BZR2	46.496	11.338	331.2	Temperate continental climate, humid summers	Valley
	GENO	44.419	8.921	153.5	Mediterranean climate, wet winters, and dry summers	Coastal and mountainous terrain
	BOR1	52.277	17.073	124.9	Temperate continental climate, with cold winters and hot summers	Hilly terrain
	HKSL	22.372	113.928	95.3	Subtropical monsoon climate with marine characteristics	Low mountain and hilly area
	JFNG	30.516	114.491	71.3	Subtropical monsoon climate	Flat terrain with scattered low hills in the periphery
	HKWS	22.434	114.335	63.8	Subtropical monsoon climate with marine characteristics	Coastal hilly terrain
Low-altitude plains/ Coastal areas	FFMJ	50.091	8.665	178.3	Where maritime and continental influences converge, with frequent meteorological changes	Plain City
	BRUX	50.798	4.359	158.3	Temperate maritime, with year-round humidity	Plain City
	BOGI	52.475	21.035	139.9	Temperate continental climate, humid summers	Flat terrain
	WSRT	52.915	6.604	86	Temperate maritime, with stable weather conditions	Lowland Plain
	MEDI	44.520	11.647	50	humid subtropical climate	Flat alluvial plain
	KMNM	24.464	118.389	49.1	Subtropical monsoon climate	Coastal plain of southern Fujian, flat terrain
	WUH2	30.532	114.357	28.2	Subtropical monsoon climate	Central Jianghan Plain, low-lying and flat terrain

**Table 2:** PPP data processing strategy.

Parameter	Strategy	
Data Processing	Observation types	Ionospheric-free combination
	Solution mode	Static
	Frequency	BDS-3:B1I/B3I GPS:L1/L2 GLONASS:L1/L2 Galileo: E1/E5a
	Sampling interval	30 s
	Elevation cutoff	10°
Error Correction	Satellite orbit/clock offset	IGS orbit/clock products
	Ionospheric delay	IF combination
	Tropospheric delay	Saastamoinen
	Phase correction	Corrected by the model
	Satellite attitude	Nominal model
	Antenna phase centre	igs14_igs.atx
Parameter Estimation	Receiver coordinates	Fixed as a priori values
	Receiver clock bias	White noise
	Tropospheric wet delay	Estimated as a random walk process noise; Mapping function: VMF3
	Ambiguity	Floating-point solution

**Table 3:** Comparison of parameter settings for experimental models.

Forecasting Model	Speciality	Optimal Parameter Settings
CNN-ATT	CNN extracts temporal-spatial local features Attention focuses on key positions	Conv kernel size = 3, hidden size = 32, dropout = 0.1, lr = 0.001 [16,25]
Informer	ProbSparse Attention is suitable for long sequence prediction.	encoder = 2, d_model = 64, learning rate = 0.0005, dropout = 0.05 (Zhou et al., 2021) [26]
TimesNet	Reconstructing one-dimensional time series into two-dimensional tensors enables precise extraction of distinct frequency features through stacked Fourier Mix Blocks.	Epochs = 50, patience = 3, learning rate = 0.001, batch size = 16, d_model = 32, d_ff = 32, top_k = 5, conv kernel num = 6, dropout = 0.1

**Table 4:** Statistics of PPP ZTD accuracy at each station.

Station	BRUX	WSRT	OBE4	BOR1	FFMJ	GANP	GOP6
Accuracy/mm	3.46	3.89	4.02	4.12	44.09	4.55	4.84
Station	GENO	GRAC	ZIM2	REDU	BZR2	BOGI	MEDI
Accuracy/mm	5.0	4.35	3.85	4.15	4.05	3.60	3.95
Station	URUM	JFNG	WUH2	HKSL	HKWS	KMNM	
Accuracy/mm	5.36	4.03	3.56	3.99	4.32	4.65	

**Table 5:** The RMSE and MAE of the predicted ZWD for each season by three models in high-altitude mountainous areas.

Station Name	Season	CNN-ATT		Informer		TimesNet	
		RMSE/mm		MAE/mm	RMSE/mm	RMSE/mm	MAE/mm
GRAC	Spring	8.5	7.2	6.1	5.4	**5.0**	**4.9**
	Summer	11.6	9.0	8.2	7.6	**5.6**	**5.4**
	Autumn	9.8	8.5	7.0	6.6	**5.3**	**5.1**
	Winter	7.5	6.6	5.5	4.6	**4.8**	**4.6**
ZIM2	Spring	8.2	7.7	6.8	6.2	**5.7**	**4.9**
	Summer	11.6	10.5	8.1	7.8	**7.8**	**7.4**
	Autumn	9.5	8.8	7.5	6.5	**6.6**	**6.5**
	Winter	7.4	6.3	6.3	5.6	**5.5**	**5.3**
URUM	Spring	8.0	6.9	7.8	6.7	**5.5**	**5.2**
	Summer	9.2	8.9	9.6	8.5	**6.7**	**6.7**
	Autumn	8.7	7.6	8.4	8.0	**5.9**	**4.9**
	Winter	7.5	6.8	7.9	6.9	**5.1**	**4.9**
GANP	Spring	7.6	7.0	6.1	5.5	**5.2**	**5.1**
	Summer	9.3	8.8	7.6	7.0	**5.9**	**5.6**
	Autumn	8.5	7.9	6.7	6.3	**5.6**	**5.2**
	Winter	7.0	6.8	5.9	4.8	**5.2**	**5.0**
OBE4	Spring	7.9	7.1	6.7	5.6	**5.5**	**5.0**
	Summer	9.4	9.2	8.9	7.1	**6.1**	**5.8**
	Autumn	8.6	8.1	7.0	6.6	**5.5**	**5.1**
	Winter	6.8	5.9	6.4	5.7	**5.0**	**4.8**
GOP6	Spring	7.9	7.5	6.5	5.5	**5.4**	**5.5**
	Summer	10.9	9.9	7.8	6.7	**6.1**	**5.6**
	Autumn	8.5	7.5	7.0	6.5	**5.8**	**5.4**
	Winter	7.3	6.4	5.4	4.2	**5.4**	**5.0**

The bolded font in the table indicates the model accuracy with the best predictive performance at that observation station.

**Table 6:** The RMSE and MAE of the predicted ZWD for each season by three models in the mid-altitude hilly regions.

Station Name	Season	CNN-ATT		Informer		TimesNet	
		RMSE/mm		MAE/mm	RMSE/mm	RMSE/mm	MAE/mm
REDU	Spring	8.3	6.7	6.9	5.0	**4.7**	**4.6**
	Summer	11.6	7.9	8.7	6.6	**4.4**	**4.1**
	Autumn	9.3	7.6	7.6	5.7	**5.0**	**4.9**
	Winter	7.4	6.0	5.8	4.6	**4.7**	**4.5**
BZR2	Spring	8.6	6.5	6.7	5.1	**4.9**	**4.8**
	Summer	10.7	8.0	8.4	6.5	**4.7**	**4.1**
	Autumn	9.2	7.3	7.4	5.8	**5.1**	**5.0**
	Winter	7.6	5.7	5.9	4.6	**4.9**	**4.5**
GENO	Spring	8.3	7.2	5.7	4.8	**5.0**	**4.5**
	Summer	10.5	9.5	5.8	5.1	**5.4**	**4.8**
	Autumn	9.3	6.9	6.4	5.6	**5.3**	**4.7**
	Winter	7.2	5.3	5.5	4.8	**4.7**	**3.7**
BOR1	Spring	8.4	7.3	6.7	5.3	**5.2**	**4.8**
	Summer	9.7	9.2	8.4	8.0	**5.7**	**5.1**
	Autumn	9.2	8.7	7.4	5.6	**5.5**	**5.0**
	Winter	7.4	6.0	5.9	4.4	**4.9**	**4.3**
HKSL	Spring	8.5	6.6	6.9	5.2	**5.1**	**4.8**
	Summer	9.9	8.6	8.5	6.3	**5.9**	**5.1**
	Autumn	9.5	7.9	7.1	5.9	**5.4**	**5.0**
	Winter	7.1	6.2	6.0	4.5	**5.1**	**4.6**
JFNG	Spring	8.7	7.1	6.4	5.3	**4.8**	**5.0**
	Summer	11.9	8.6	8.8	6.4	**4.7**	**4.4**
	Autumn	9.7	7.5	7.1	5.5	**5.4**	**5.1**
	Winter	7.2	6.3	6.1	4.2	**4.5**	**4.7**
HKWS	Spring	8.2	6.0	5.8	4.4	**5.1**	**4.6**
	Summer	10.7	7.8	5.3	4.9	**5.4**	**4.9**
	Autumn	9.2	7.2	6.0	5.1	**5.3**	**4.8**
	Winter	7.8	5.7	5.9	3.7	**4.8**	**4.3**

The bolded font in the table indicates the model accuracy with the best predictive performance at that observation station.

**Table 7:** The RMSE and MAE of the predicted ZWD for each season by three models in the low-altitude plains/coastal regions.

Station Name	Season	CNN-ATT		Informer		TimesNet	
		RMSE/mm		MAE/mm	RMSE/mm	RMSE/mm	MAE/mm
FFMJ	Spring	7.6	6.3	6.6	4.5	**5.7**	**5.2**
	Summer	8.4	7.8	7.5	5.4	**6.5**	**5.5**
	Autumn	8.4	7.1	7.3	5.2	**6.0**	**5.4**
	Winter	6.6	5.5	5.5	4.0	**5.4**	**5.0**
BRUX	Spring	7.4	6.2	6.1	4.6	**5.0**	**4.6**
	Summer	7.8	7.0	7.2	5.5	**5.3**	**4.9**
	Autumn	8.2	7.0	6.7	5.3	**5.3**	**4.8**
	Winter	6.4	5.4	5.4	3.9	**4.7**	**4.4**
BOGI	Spring	7.7	6.2	6.3	4.8	**4.9**	**4.6**
	Summer	8.5	7.6	7.1	5.8	**4.9**	**4.7**
	Autumn	8.7	7.2	7.0	5.5	**5.2**	**4.8**
	Winter	6.5	5.6	5.4	4.1	**4.6**	**4.4**
WSRT	Spring	8.1	7.1	7.2	5.1	**5.1**	**4.9**
	Summer	10.5	9.4	9.7	6.5	**5.4**	**5.7**
	Autumn	9.3	8.0	7.6	5.8	**5.4**	**5.6**
	Winter	7.3	6.3	6.3	4.6	**4.8**	**5.2**
MEDI	Spring	7.3	6.1	6.3	5.0	**4.9**	**4.7**
	Summer	8.3	7.3	7.4	6.3	**5.1**	**4.9**
	Autumn	8.3	7.0	7.0	5.7	**5.2**	**4.9**
	Winter	6.5	5.5	5.5	4.3	**4.6**	**4.5**
KMNM	Spring	7.1	6.3	6.5	4.5	**5.1**	**4.7**
	Summer	8.1	7.0	7.2	5.4	**5.4**	**5.0**
	Autumn	8.5	6.7	7.0	5.6	**5.3**	**4.9**
	Winter	6.1	5.5	5.4	4.2	**4.8**	**4.5**
WUH2	Spring	8.4	7.0	7.3	5.4	**5.2**	**5.5**
	Summer	10.8	9.3	9.6	6.4	**5.5**	**5.8**
	Autumn	9.0	8.3	7.9	6.1	**5.5**	**5.7**
	Winter	7.0	6.6	6.4	4.7	**4.9**	**5.3**

The bolded font in the table indicates the model accuracy with the best predictive performance at that observation station.

## Data Availability

The data and code specifications for this study can be obtained from the corresponding literature upon the author’s request.
